# Endometrium Immunomodulation to Prevent Recurrent Implantation Failure in Assisted Reproductive Technology

**DOI:** 10.3390/ijms232112787

**Published:** 2022-10-24

**Authors:** Mustapha Benkhalifa, Fabien Joao, Cynthia Duval, Debbie Montjean, Molka Bouricha, Rosalie Cabry, Marie-Claire Bélanger, Hatem Bahri, Pierre Miron, Moncef Benkhalifa

**Affiliations:** 1 HB Laboratory, Tunis TN 1007, Tunisia; 2 Faculty of Sciences of Bizerte, University of Carthage, Bizerte TN 7021, Tunisia; 3 Fertilys Reproductive Center, Laval, QC H7S 1Z5, Canada; 4 Department of Reproductive Medicine, Reproductive Biology & Genetics, University Hospital and School of Medicine Picardie University Jules Verne, 80054 Amiens, France; 5 PeriTox Laboratory, CURS, Amiens Sud, 80480 Salouël, France

**Keywords:** infertility, assisted reproductive technology, implantation failure, endometrium immunomodulation

## Abstract

After more than four decades of assisted reproductive technology (ART) practice worldwide, today more than 60% of women undergoing in vitro fertilization (IVF) treatments fail to become pregnant after the first embryo transfer and nearly 20% of patients are suffering from unexplained recurrent implantation failures (RIFs) and repeated pregnancy loss (RPL). The literature reported different causes of RIF–RPL, mainly multifactorial, endometrial and idiopathic. RIF remains a black box because of the complicated categorization and causes of this physio-pathological dysregulation of implantation and pregnancy process after ovarian stimulation. Many options were suggested as solutions to treat RIF–RPL with controversial results on their usefulness. In this article, we reviewed different possible therapeutic options to improve implantation rates and clinical outcomes. Based on our experience we believe that endometrium immunomodulation after intrauterine insemination of activated autologous peripheral blood mononuclear cells (PBMCs) or platelet-rich plasma (PRP) can be a promising therapeutic solution. On the other hand, peripheral lymphocyte balance typing, specific cytokines and interleukins profiling can be proposed as predictive biomarkers of implantation before embryo transfer.

## 1. Introduction

In assisted reproductive technology (ART) programs, 60–70% of women fail to become pregnant after embryo transfer. Repeated implantation failure (RIF) remains a black box in daily practice due to the complicated categorization and causes of this physio-pathological dysregulation [1]. Different causes of RIF were reported, mainly multifactorial, endometrial and idiopathic. Multifactorial RIF can be caused by maternal and paternal factors, gamete and embryo quality, infections and lifestyle changes in combination with psychological status and oxidative stress [1,2]. Impaired endometrium function such as abnormal growth or loss of vascularization can account for endometrial RIF, but idiopathic RIF, caused mainly by abnormal cross-talk between the embryo and endometrium, remains the principal question and needs to be elucidated [1].

RIF may be defined as a failure to obtain a pregnancy after multiple viable embryo transfers during IVF treatment [3], but its definition is inconsistent between studies. The most common definition was portrayed by Bashiri and colleagues [4] who describe RIF as three or more pregnancy failures following the transfer of at least three good-quality embryos [4]. However, other authors such as Coughlan and colleagues [5] suggest including maternal age, number of embryos transferred and number of previous cycles to the definition of RIF [5]. Interestingly, a consensus is emerging thanks to a recent extensive survey. It was proposed to define RIF as the failure to achieve a clinical pregnancy after 2–3 IVF cycles with 1–4 good-quality embryos [6]. RIF is a challenge for clinicians as its etiology includes various possible causes [2].

The causes of RIF can be divided into two categories: maternal (uterine anatomic abnormalities, chronic endometritis, non-receptive endometrium, antiphospholipid antibody syndrome and immunological factors) and embryonic (genetic defects and other factors specific to embryonic development) causes [3]. In the absence of male factors, oxidative stress, bad-quality embryos and anatomical abnormalities such as hydro-salpinx and thrombophilia, RIF seems to be caused by impaired endometrial function such as abnormal endometrial growth or loss of vascularization [4]. However, RIF caused by immunological factors could be manageable using several innovative therapeutic options. Among them, intrauterine administration of human chorionic gonadotropin (HCG), granulocyte colony-stimulating factor (G-CSF) or autologous peripheral blood mononuclear cells (PBMCs) has been suggested as a treatment for patients suffering from RIF [4,7,8,9,10,11,12,13,14,15,16].

Intrauterine administration of autologous PBMC prior to embryo transfer was proposed to regulate the immune environment of the endometrial tissue [4]. In 2006, Yoshioka and colleagues were the first to propose this immunotherapy to help RIF patients [7]. Since then, this therapeutic option was recommended as an effective treatment for RIF according to numerous studies [4,7,8,9,10,11,12,13,14]. The present study is a review aiming at summarizing studies that used this immunotherapy to evaluate its benefit regarding RIF patients.

## 2. Endometrium Immunomodulation via Intrauterine Insemination of Activated Autologous Peripheral Blood Mononuclear Cells (PBMCs) 

**Figure 1:**
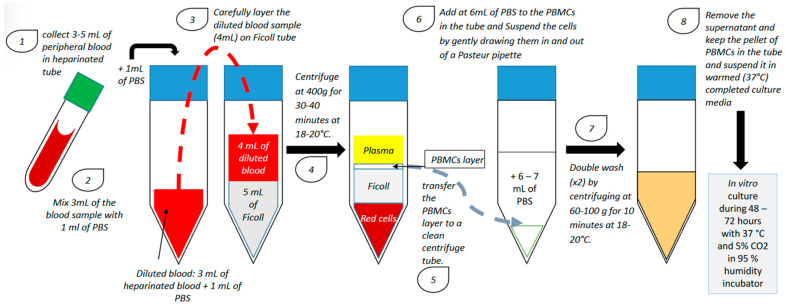
PBMC isolation technique and in vitro culture (PBS: phosphate-buffered saline; PBMC: peripheral blood mononuclear cell).

PBMCs from patients with RIF are usually isolated during the ovulation period using a lymphocyte separation medium composed of an iso-osmotic poly-sucrose and sodium diatrizoate solution to separate mononuclear cells (including B-lymphocytes, T-lymphocytes and monocytes) from the other blood cells. After separation, PBMCs are generally activated with hCG or corticotropin-releasing hormone (CRH) and cultured in vitro for 24–72 h in a humidified incubator with 5% CO_2_ at 37 °C (Figure 1). 

After culture, PBMCs are administered in utero using a catheter [4,7,8,9,10,11,12,13,14,15]. However, the number of cells administered in utero is not homogeneous among all studies investigating the use of PBMC in the treatment of RIF (Table 1). Although there were some methodological variations between studies in terms of the number of previous cycles, cycle type, and number and quality of transferred embryos, patients were generally administered with 10 to 30 million PBMCs [7,8,9,10,11,12,13,14,15,16]. Madkour and colleagues showed a significant increase in clinical pregnancy rate (CPR) with only 1 million cells [10]. Furthermore, in a recent meta-analysis, Qin and colleagues have demonstrated that CPR was higher when less than 100 million PBMCs/mL were administered in utero, suggesting that although the quantity of cells inseminated is not homogeneous, intrauterine administration of PBMC does appear to be an effective treatment for patients suffering from RIF [17].

## 3. Immunoregulation of the Endometrium during Embryo Implantation: Biological Function and Molecular Pathway

To achieve successful embryo implantation and pregnancy, an appropriate dialogue between the embryo and the endometrium must take place [18].

In the uterine environment, a particular form of natural killer (NK) cells with a unique transcriptional profile, the uterine NK (uNK) cells, represents the most abundant lymphocyte population, especially in the endometrium [19,20,21]. In fact, most of the immune cells present in the uterus usually display a unique phenotype [18]. Peripheral blood NK cells express CD56^+^CD16^+^ at their membrane surface and are characterized by a highly cytotoxic profile [22]. However, uNK cells are less toxic since they do not express CD16 on their membrane surface [23]. During the menstrual cycle, levels of uNK cells start to increase in the mid-secretory phase, which could explain their importance in embryo implantation [24,25,26,27]. 

Dendritic cells (DCs), another type of innate immune cells, have a crucial role in the site of embryo implantation and maternal–fetal interface. DCs act as antigen-presenting cells to T cells and have the unique ability to induce a primary immune response, a phenomenon crucial for successful pregnancy [28]. In addition, DCs can influence trophoblast invasion by regulating the secretion of cytokines and the production of endometrial cell-surface proteins. Through the regulation of immune cell functions and actions, DCs have a major role in the establishment of a special local immune environment essential for embryo implantation and placental development [29]. Human decidual DCs, however, seem to have an immature phenotype characterized by a low expression of CD40, CD80, CD86 and CD205 [30,31]. DCs seem to be involved in the immune tolerance of the implantation site through the regulation of T-cell proliferation and the elimination of antigen-specific T cells. In the decidua, uterine dendritic cells (uDCs) are also crucial in maintaining pregnancy [32]. Since the 1990s, it has been known that maternal T cells are essential to the complex mechanisms of immune tolerance, a phenomenon critical to the invasion of the endometrium by the blastocyst [33].

T-cell interactions can be performed directly by cell–cell contact or indirectly through the secretion of pro-inflammatory or anti-inflammatory cytokines [34]. Pro-inflammatory cytokines such as interleukin (IL)-1β, -6, -12, -2 and -18; tumor necrosis factor alpha (TNF-α) and interferon gamma (IFN-γ) are mainly produced by T helper (Th) 1 cells, while anti-inflammatory cytokines such as IL-4, IL-10, IL-13 and TGF-β1 are mostly secreted by Th2 cells [35]. The pro-inflammatory Th1 profile was shown to be associated with successful and normal pregnancy at early and late pregnancy stages. In the midgestation stage, however, a shift to an anti-inflammatory Th2 profile must take place to establish tolerance to the foreign fetal antigens [36]. An imbalance in these cytokine profiles has been associated with spontaneous abortion and common complications of pregnancy [37,38,39]. Moreover, it has been shown that levels of pro-inflammatory cytokines (such as IL-2 and IFN-γ) decreased while levels of anti-inflammatory cytokines (such as IL-4 and IL-10) increased in the induction of immune tolerance to allografts [40,41]. The implication of T cells, especially CD4+ CD25+ Foxp3+ Treg cells, in the initial stages of pregnancy is therefore needed for the prevention of an alloreactivity action by the endometrium against the fetus through cascades of immunoregulation actions [42,43]. 

Treg, Th1 and Th2 cells are, however, not the only T-cell subtypes known to be crucial for successful embryo implantation. Th17 cells, a subset of T cells showing remarkable plasticity, are also indispensable in the immunoregulation of embryo implantation as well as in maintaining normal pregnancy [44].

Monocytes and macrophages also play an important role during the menstrual cycle and pregnancy [14,45,46]. Macrophages regulate trophoblast activity by promoting endometrial tissue remodeling and angiogenesis [47]. Pregnancy hormones directly and indirectly modulate the recruitment of monocytes in the uterus and participate in their differentiation and stimulation into functional macrophages [48]. Intrauterine administration of PBMCs could also be a source of hCG-activated macrophages and regulate the uterine environment at the embryo implantation site [14].

## 4. Endometrium Immunomodulation with Activated PBMCs and Embryo Implantation

Intrauterine administration of PBMCs in patients suffering from RIF aims to improve endometrial receptivity by regulating the Th1/Th2 cytokine ratio and growth factors to stimulate many cascades of cytokines and matrix metalloproteinase actions [1,7,10,15]. Increased peripheral blood Th1/Th2 ratio was shown to be detrimental to embryo implantation [39]. However, PBMCs produce many cytokines that can regulate Th1/Th2 imbalance in women suffering from RIF [39]. Furthermore, PBMCs are known to increase the secretion of growth factors and Th1 pro-inflammatory and anti-inflammatory cytokines at the time of embryo implantation to boost endometrial receptivity [4,9,10,11]. This immunotherapy was shown to improve progesterone (P4) production in cultured human granulosa luteal cells [49]. Ovarian steroids such as P4 and β-hCG are among the most crucial factors needed in the immunoregulation of embryo implantation [50]. Luteinizing hormone (LH) and hCG have also an important role in establishing the immune tolerance mechanisms of embryo implantation. These two gonadotropins were shown to affect immune cells by binding to the LH/hCG receptors present at the surface of several immune cell types [50]. Furthermore, it has been shown that hCG has the capacity to downregulate pro-inflammatory immune responses during pregnancy [51]. During the embryo implantation window, β-hCG seems to play a role in the immunoregulation of the endometrium in increasing Fas ligand expression (APO-1, CD95) in the endometrial cells to facilitate trophoblast invasion [52]. Increased peripheral blood Treg cell levels have also been shown to be positively associated with higher pregnancy rates in IVF treatment [53]. These cells being attracted to trophoblasts by hCG [51] supports the fact that the administration of hCG could be an effective treatment for some infertile women. Moreover, it has been shown by Mansour and colleagues that intrauterine hCG injection before embryo transfer could significantly improve implantation and pregnancy rates [54].

Intrauterine administration of PBMCs for patients suffering from RIF has been shown to be specifically efficient for increasing implantation and pregnancy rates in women with three or more previous implantation failures [17,55]. Recently, Nobijari and colleagues and Pourmoghadam and colleagues presented a different strategy to administer PBMCs in RIF patients using frozen–thawed embryo transfers [14,15]. Nobijari and colleagues confirmed the effectiveness of this immunotherapy by showing an increase in CPR in patients with three or more implantation failures undergoing frozen–thawed embryo transfer [15]. Pourmoghadam and colleagues only administrated PBMCs in utero in RIF patients with a low Th-17/Treg cell ratio [14]. Furthermore, in the study of Pourmoghadam and colleagues, PBMCs were activated in vitro with 10 IU/mL hCG for 48 h before the intrauterine administration, while Nobijari and colleagues activated the PBMCs in vitro with CRH for 48–72 h [14,15]. In RIF patients, it has also been shown that levels of IL-1β, TNF-α and IFN-γ, three pro-inflammatory cytokines, were increased in the PBMC culture medium, suggesting that PBMCs secrete these Th1 cytokines when treated with hCG [14]. Moreover, Pourmoghadam and colleagues have shown that CPR and live birth rates increased significantly and miscarriage rates decreased significantly in RIF patients treated with PBMCs compared to control [14]. In addition, Makrigiannakis and colleagues have shown that the insemination of autologous PBMCs treated with CRH before blastocyst or early cleaved embryo transfer presented better results than PBMCs without CRH treatment in terms of CPR in women with RIF [13].

Therefore, these three studies supported the effectiveness of this immunotherapy for patients suffering from RIF undergoing fresh or frozen–thawed embryo transfer, especially when PBMCs are treated with CRH [13,14,15]. However, these findings are still limited because, in the study of Pourmoghadam and colleagues for example, the authors measured only three pro-inflammatory cytokines, and they did not show anti-inflammatory cytokine levels with PBMC administration for RIF women or in a control group [14]. The increase in these cytokine levels should be compared to a control, not treated cells, but the authors did not perform this comparison [14].

## 5. Other Endometrium Immunomodulation Options

Immunological therapy approaches other than intrauterine administration of PBMCs for the management of RIF patients were reported in the literature. These immunotherapies focus on elevated Th1/Th2 ratio, abnormal TNF-α/IL-10 ratio, elevated NK cells and auto-antibodies. One of these immunomodulatory agents that have been described for RIF patients is intravenous immunoglobulin IgG (IVIg). Patients receiving this treatment have shown significantly higher implantation and clinical pregnancy rates compared to non-treated patients [56]. This treatment has been extensively used, but the results are heterogeneous [57,58,59,60]. According to many studies, the application of IVIg has shown positive effects on RIF patient pregnancy rates and in patients with increased immunological risk factors [24,61,62,63,64].

Granulocyte colony-stimulating factor (G-CSF) was also shown to have positive effects on embryo implantation in women suffering from RIF, especially when endometrial thickness was insufficient [65]. Furthermore, a recent meta-analysis showed that G-CSF was an effective treatment for women with thin endometrium or with recurrent IVF failures [66]. G-CSF was originally used as a treatment for thin endometrium to thicken it. Increased implantation rates were shown after G-CSF treatment in patients with an endometrium thickness ≥7 mm on the day of embryo transfer [67]. These results were confirmed by another study conducted by Xu and colleagues in 2015 in which they showed a higher implantation rate in women treated with G-CSF compared to controls [68]. Furthermore, Kalem and colleagues have shown that the administration of G-CSF into the uterine cavity in RIF patients with normal endometrium did not alter the endometrial thickness, clinical pregnancy rates or live birth rates in comparison with a control group [69].

Vitamin E, which has been shown to improve capillary blood flow in different organs [70,71], and sildenafil citrate (Viagra), which improves uterine artery blood flow [72,73], were also proposed as a treatment for thin endometrium [72,73,74]. In the study of Miwa and colleagues, 23 out of 25 patients showed improved radial artery, 17 patients had increased endometrial thickness and 13 patients developed an endometrium thickness of more than 8 mm [74]. Sher and Fisch were the first to suggest the use of sildenafil during the follicular phase and until ovulation trigger as a treatment for thin endometrium of women undergoing IVF with fresh embryo transfer [72]. They reported an improvement in uterine blood flow and in endometrial thickness [72]. These results were confirmed in a larger cohort study showing a 45% pregnancy rate [73]. However, a randomized controlled trial study reported no significant difference in endometrial thickness and pregnancy rate after administration of sildenafil and valerate estradiol during the luteal phase following fresh embryo transfer [75]. Another randomized controlled trial study did not show any improvement in uterine blood flow or in endometrial thickness [76]. Recently, a randomized placebo-controlled trial study reported that the use of vaginal sildenafil on the hCG injection day did not present a statistically significant improvement in endometrium thickness; however, the implantation (chemical pregnancy) was significantly higher in women who received sildenafil with placebo compared to women who received only sildenafil or only placebo [77]. More trials are needed to confirm the effectiveness of these treatments on endometrium thickness and/or RIF.

In 2015, Nakagawa and colleagues proposed a treatment using immunosuppressive drugs such as tacrolimus, one of the major immune-suppressive agents that have been used after allogeneic organ transplantation to reduce the alloreactivity of a recipient’s immune system and to decrease the risk of the rejection [78,79]. This treatment has shown positive results on successful implantation and pregnancy outcome in RIF patients with elevated Th1/Th2 ratios, suggesting that this immunological imbalance plays a crucial role in causing RIF [78]. However, the posology of this drug must be determined more accurately to maintain the levels of the essential Th1 cytokines necessary for embryo implantation [39].

Another treatment using atosiban administration was proposed for RIF women. In fact, atosiban is a receptor of oxytocin and V1a vasopressin, proposed to avoid uterine contractions during embryo transfer, which could be detrimental in embryonic apposition [80]. However, according to the review of Makrigiannakis and colleagues, various randomized controlled trial studies reported a non-significant effect on reproductive outcomes [81,82,83,84,85], and only two non-randomized studies on RIF patients report a significant benefit after atosiban treatment [86,87]. Therefore, more randomized studies are needed to verify the efficiency of atosiban as a benefic treatment for RIF women.

In 2015, Chang and colleagues reported that autologous platelet-rich plasma (PRP) promotes endometrial growth and improves pregnancy outcomes during IVF [88]. After being collected from the peripheral vein in acid citrate dextrose solution A (ACD-A) anticoagulant tubes, PRP was prepared by separating the various components of the blood using multiple centrifugations [89]. This PRP, within 10 min after clotting, can activate cytokines and growth factors which become bioactive and increasingly secreted. These factors include vascular endothelial growth factor (VEGF), transforming growth factor (TGF), platelet-derived growth factor (PDGF) and epidermal growth factor (EGF), which can regulate cell migration, attachment, proliferation and differentiation, while promoting extracellular matrix accumulation [90]. This could lead to ameliorated implantation conditions and improved pregnancy, as was revealed by Chang and collaborators [88]. Other studies could confirm these results; for example, in 2019, Kim et al. showed that autologous PRP treatment increases the activity of cytokines and growth factors compared to that observed without the use of PRP, especially when combined with frozen–thawed embryo transfer [91]. These studies support the suggestion of PRP as a useful treatment for RIF. However, in a recent study that used PRP treatment in patients with a history of failed implantation before frozen–thawed embryo transfer, the authors did not find significant differences in the pregnancy results in comparison with controls [92]. A recent study by Ibañez-Perez and colleagues suggested a non-invasive method of microRNA-based signatures obtained from very small volumes of endometrial fluid collected just before day 5 frozen embryo transfers to identify the competence of the endometrium in implantation [93]. This technique could help physicians to avoid RIF by changing the embryo transfer strategy when the results show an unfavorable implantation pattern by using immunomodulation techniques from the first IVF cycle [93].

## 6. Conclusions

There is no scientific consensus about the best immunological treatment for RIF patients presenting an imbalanced Th1/Th2 ratio or immune dysregulation. However, recent studies have shown the potential of the intrauterine administration of hCG-activated PBMCs and activated PRP as a good way to modulate endometrial receptivity. The immunotherapy field strategy needs to be further elucidated for a better understanding of maternal immunotolerance to embryo implantation. Proteomic investigations of biomarkers produced by immunological cells and their pathways should be continued to identify the exact combination of immunological factors needed for successful implantation. Correcting immunological dysregulations in embryo implantation by intrauterine administration of PBMCs or treatment with activated PRP seems to be a promising solution in RIF. It is clear that we need to know much more about maternal immune tolerance and the exact role of each biomarker involved in embryo–endometrium cross-talk to improve implantation and reduce repeated implantation failure and pregnancy loss. 

## Figures and Tables

**Table 1:** Main studies using PBMCs to treat RIF.

Study	Number of Previous Failed IVF Cycles	Sample Size		Day of Blood Collection	PBMCs Co-cultured with	Duration of PBMC Culture	Number of PBMCs Administered In Utero	Transfer Type	Stage of Embryo	Implantation Rate (Control vs. Case)	Clinical Pregnancy Rate (Control vs. Case)	Miscarriage Rate (Control vs. Case)	Live Birth Rate (Control vs. Case)
		Control	Case										
Yoshioka et al., 2006 [7]	≥4	18	17	On the day of oocyte retrieval	hCG: 5 IU/mL	48 h	20 × 10^6^	Fresh	1, 2 or 3 blastocysts	4.1% vs. 23.4% (*p* = 0.0034)	11.1% vs. 41.2% (*p* = 0.042)	Not specified	7.6% vs. 55.6% (*p* = 0.013)
Okitsu et al., 2011 [16]	≥1	170	83	On the day following ovulation or the day after	Not activated	No culture	30 × 10^6^	Frozen/ thawed	early cleavage embryo or blastocyst	≥1 RIF: 21.1% vs. 21.6% (ns); 3 RIF: 9.38% vs. 25.0% (*p* = 0.041)	≥1 RIF: 32.9% vs. 34.9% (ns); ≥3 RIF: 16.7% vs. 42.1% (*p* = 0.039)	Not specified	≥ 1 RIF: 21.8% vs. 21.7% (ns); ≥3 RIF: 11.1% vs. 21.2% (ns)
Makrigiannakis et al., 2015 [9]	≥3	45	45	On the day of oocyte retrieval	CRH: 10^7^ M/1.10^6^ cells/mL	48 h	20 × 10^6^ + 10^7^ CRH	Fresh	2 or 3 blastocysts (grade 3BB and above)	Not specified	0% vs. 44.44% (*p* < 0.001)	Not specified	Not specified
Madkour et al., 2016 [10]	≥2	27	27	On the day of ovulation induction	Complete culture medium + 75 IU of hMG	72 h	1 × 10^6^	Fresh	1, 2 or 3 early cleavage embryos	≥2 RIF: 9% vs. 22% (*p* = 0.02); 2 RIF vs. ≥ 3 RIF: 15% vs. 35% (*p* = 0.09)	≥2 RIF: 15% vs. 44% (*p* = 0.045); 2 RIF vs. ≥ 3 RIF: 29% vs. 70% (*p* = 0.04)	≥2 RIF: 17% vs. 75% (*p* = 0.08) 2 RIF vs. ≥ 3 RIF: 20% vs. 14% (*p* = 0.8)	Not specified
Yu et al., 2016 [11]	≥3	105	93	On the day following ovulation	hCG: 10 IU/mL	24 h	10–20 × 10^6^	Frozen/ thawed	early cleavage embryo	11.43% vs. 23.66% (*p* < 0.05)	20.95% vs. 46.24% (*p* < 0.05)	31.8% vs. 20.9% (ns)	14.28% vs. 34.41% (*p* < 0.05)
Li et al., 2017 [12]	≥1	339	294	Two days before embryo transfer	hCG: 10 IU/mL	24 h	10–20 × 10^6^	Fresh and frozen/ thawed	2 or 3 early cleavage embryos or 2 or 3 grade 2 blastocysts at day 5 and 3BB and above at day 6	1 RIF: 32.33% vs. 29.35% (ns); 2 RIF: 27.74% vs. 35.98% (*p* = 0.048); 3 RIF: 26.23% vs. 23.20% (ns); ≥4 RIF: 4.88% vs. 22.00% (*p* = 0.014)	1 RIF: 41.23% vs. 43.75% (ns); 2 RIF: 42.18% vs. 48.15% (*p* = 0.016); 3 RIF: 36.84% vs. 42.22% (ns); ≥4 RIF: 14.29% vs. 39.58% (*p* = 0.038)	Not specified	1 RIF: 36.84% vs. 37.5% (ns); 2 RIF: 33.33% vs. 34.26% (ns); 3 RIF: 24.56% vs. 28.89% (ns); ≥4 RIF: 9.58% vs. 33.33% (*p* = 0.038)
Makrigiannakis et al., 2019 [13]	≥3	26	26	On the day of oocyte retrieval	CRH: 10^7^ M/1.10^6^ cells/mL	48 h	20 × 10^6^ + 10^7^ M CRH	Fresh	2 or 3 grade 1 or 2 early cleavage embryos	Not specified	0% vs. 57,69% (*p* < 0.01)	Not specified	Not specified
Nobijari et al., 2019 [15]	≥1	128	122	5 days before the frozen/thawed embryo transfer	CRH (concentration not specified)	48–72 h	20 × 10^6^ + 10^7^ M CRH	Frozen/ thawed	early cleavage embryo or blastocyst	Not specified	<3 RIF: 30.4% vs. 30.8% (*p* = 0.91); ≥3 RIF: 19,7% vs. 38,6% (*p* = 0.01)	Not specified	Not specified
Pourmoghadam et al., 2020 [14]	≥3	50	50	On the day of ovulation induction	hCG: 10 IU/mL daily	48 h	15–20 × 10^6^	Frozen/ thawed	early cleavage embryo or blastocyst	Not specified	22% vs. 42% (*p* = 0.032)	24% vs. 8% (*p* = 0.029)	20% vs. 38% (*p* = 0.047)

CRH: corticotropin-releasing hormone; hCG: human chorionic gonadotropin; RIF: recurrent implantation failure.
